# Case Report: Acute liver failure in systemic lupus erythematosus with autoimmune hepatitis–primary biliary cholangitis overlap: a belimumab-containing regimen and longitudinal follow-up

**DOI:** 10.3389/fimmu.2026.1915385

**Published:** 2026-10-06

**Authors:** Lina Zhang, Tong Zhang, Jing Zhang, Xiuhong Wang, Yihui Rong, Sheng-Guang Li

**Affiliations:** 1 Department of Rheumatology and Immunology, Peking University International Hospital, Beijing, China; 2 Department of Pathology, Peking University International Hospital, Beijing, China; 3 Department of Pathology, China-Japan Friendship Hospital, Beijing, China; 4 Department of Infectious Diseases, Peking University International Hospital, Beijing, China

**Keywords:** autoimmune hepatitis, BAFF, belimumab, liver injury, overlap syndrome, primary biliary cholangitis, systemic lupus erythematosus

## Abstract

**Background:**

Severe autoimmune liver injury as the first manifestation of systemic lupus erythematosus (SLE) is rare, and concomitant autoimmune hepatitis–primary biliary cholangitis (AIH–PBC) overlap is uncommon. B-cell-activating factor (BAFF) provides a biological rationale for adjunctive therapy, although clinical evidence remains limited.

**Case presentation:**

A 72-year-old woman presented with 4 weeks of fatigue and anorexia and developed coagulopathy (international normalized ratio [INR] 1.8), ascites, and hepatic encephalopathy. Autoimmune testing showed antinuclear antibody 1:1280, high-titer anti-double-stranded DNA antibodies with hypocomplementemia, immunoglobulin G (IgG) 30.0 g/L, and antimitochondrial antibody positivity. Hepatitis B surface antigen, hepatitis C antibody, hepatitis E antibody, and Epstein–Barr virus and cytomegalovirus DNA tests were negative. Liver biopsy 42 days after corticosteroid initiation demonstrated interface hepatitis, destructive small-bile-duct injury, plasma cell-rich inflammation, and Batts–Ludwig stage 2 fibrosis without cirrhosis, supporting a clinicopathologic diagnosis of AIH–PBC overlap. Previously unrecognized chronic autoimmune liver disease could not be excluded. Prednisolone was started at 50 mg/day, followed 10 days later by intravenous belimumab, with concomitant ursodeoxycholic acid. Tacrolimus was added during maintenance. By 10 October 2025, liver biochemistry had improved, IgG was 9.42 g/L, and prednisolone had been reduced to 2.5 mg/day. Liver stiffness decreased from 21.5 to 9.7 kPa.

**Conclusion:**

Sustained biochemical improvement occurred during a belimumab-containing multidrug regimen. This observation does not establish the independent efficacy of belimumab or histological regression of fibrosis.

## Introduction

Autoimmune liver diseases (AILDs) comprise autoimmune hepatitis (AIH) and primary biliary cholangitis (PBC), which may coexist as overlap syndromes characterized by mixed hepatitic and cholestatic features and often require combined ursodeoxycholic acid and immunosuppression (1). AILDs may coexist with systemic autoimmune diseases and can be difficult to distinguish from non-specific hepatic involvement in systemic lupus erythematosus (SLE) (2).

Mild liver test abnormalities are not uncommon in SLE. In contrast, acute liver failure is an exceptional presentation. Reviews of hepatic involvement in SLE suggest that liver abnormalities are usually mild, transient, and often related to drugs or non-SLE liver diseases rather than lupus itself (2). In a Mayo Clinic review of 40 SLE patients with abnormal liver enzymes, serious liver disease, including portal hypertension, cirrhosis, or hepatic encephalopathy, was rare and was usually associated with coexisting liver disorders such as AIH, viral hepatitis, or non-alcoholic fatty liver disease (3). A recent retrospective study of SLE patients with liver failure further highlighted the diagnostic heterogeneity of this presentation and reported that AIH may be diagnosed concurrently with SLE in some patients (4). These findings support a structured evaluation when SLE presents with severe hepatitis or liver failure, rather than attributing the injury to lupus hepatitis alone.

Published descriptions of SLE with AIH-PBC overlap remain sparse. The available literature consists mainly of isolated case reports or letters, conference abstracts, and small retrospective cohorts (5–10). One case report described PBC/AIH overlap developing in a patient with SLE (5). A more recent letter reported AIH-PBC overlap in an SLE patient receiving long-term B-cell depletion therapy (6). Related conference abstracts have described complex overlap syndromes in SLE, including decompensated cirrhosis or additional autoimmune cholangiopathy (7, 8). Retrospective studies also suggest that autoimmune liver disease occurs in only a minority of SLE patients, and biopsy-confirmed overlap phenotypes remain uncommon (9, 10). Therefore, liver biopsy is particularly important when severe hepatitis, cholestatic autoantibodies, hypergammaglobulinemia, or hepatic decompensation is present.

B-cell activation provides a plausible mechanistic link between SLE and autoimmune liver disease. Polyclonal B-cell activation is a hallmark of SLE and may also contribute to subsets of AILD. B-cell-activating factor (BAFF) promotes autoreactive B-cell survival, providing a rationale for investigating BAFF blockade (11, 12). Belimumab has shown preliminary benefit in difficult-to-treat AIH, but clinical evidence remains limited (12). Here, we report severe acute autoimmune liver dysfunction as the first manifestation of SLE, with biopsy findings supporting AIH–PBC overlap and sustained biochemical improvement during a belimumab-containing immunosuppressive regimen.

## Case presentation

A 72-year-old woman with no known chronic liver disease presented with 4 weeks of progressive fatigue and anorexia, followed by intermittent fever (maximum temperature 38.2 °C). Laboratory testing showed peak aspartate aminotransferase (AST) 1706 U/L, alanine aminotransferase (ALT) 317 U/L, gamma-glutamyltransferase (GGT) 261 U/L, total bilirubin 45.1 μmol/L, an international normalized ratio (INR) of 1.8, hypoalbuminemia (albumin 32.5 g/L), and a platelet count of 70 × 10^9^/L. Past medical history included photosensitivity and hypertension. Annual health examinations had not identified liver-test abnormalities, and there was no known cirrhosis or previous hepatic decompensation. She denied alcohol use. She had polyarthralgia with morning stiffness and mild scleral icterus, without rash, lymphadenopathy, peripheral edema, or hepatosplenomegaly. Vital signs were stable (temperature 37.2 °C, blood pressure 139/72 mmHg). Grade II hepatic encephalopathy was present on admission.

Initial laboratory evaluation showed systemic inflammation and cytopenias: C-reactive protein 19.23 mg/L and thrombocytopenia (platelets 70–85 × 10^9^/L). Coagulation testing showed prothrombin activity 44%, INR 1.8, and fibrinogen 141 mg/dL. Hepatitis B surface antigen (HBsAg), hepatitis C antibody (anti-HCV), and hepatitis E antibody were negative, as were Epstein–Barr virus (EBV) DNA and cytomegalovirus (CMV) DNA. Long-term amlodipine use preceded the illness by several years. No newly introduced suspected hepatotoxic drug was identified before the liver-test abnormalities. Severe acute respiratory syndrome coronavirus 2 (SARS-CoV-2) infection and the subsequent use of antipyretics and nirmatrelvir/ritonavir occurred after the onset of hepatitis. Abdominal ultrasound showed preserved liver size and morphology, a smooth surface, mildly coarse parenchymal echotexture, and a small volume of ascites.

Autoimmune profiling revealed a strongly positive antinuclear antibody (ANA) (1:1280, homogeneous) with high-titer anti-double-stranded DNA antibodies (anti-dsDNA) (673.99 IU/mL), anti-Smith antibody (anti-Sm) positivity, and anti-nucleosome/anti-histone antibodies. Complement was consumed (complement component 3 [C3] 0.39 g/L). Marked polyclonal hypergammaglobulinemia was present (immunoglobulin G [IgG] 30.02 g/L; immunoglobulin A [IgA] 6.51 g/L), and serum immunoglobulin G4 (IgG4) reached 7.21 g/L; immunofixation electrophoresis showed no monoclonal band. Antimitochondrial M2 antibody (AMA-M2) was positive (36.9 RU/mL), and smooth muscle antibody (SMA) was reported negative. Antiphospholipid antibodies were detected (anti-phosphatidylserine/prothrombin [aPS/PT] IgG 45.28 U; anti-β2-glycoprotein I [β2-GPI] IgG 28.5 CU). No associated thrombotic event was reported; antibody positivity alone did not establish antiphospholipid syndrome.

The combination of acute severe hepatitis, coagulopathy (INR >1.5), and hepatic encephalopathy was clinically consistent with an acute liver failure presentation. However, the subsequent finding of stage 2 fibrosis indicated underlying structural liver injury, so the absence of previously abnormal liver tests did not exclude an acute presentation of previously unrecognized chronic autoimmune liver disease. A history of photosensitivity and concurrent inflammatory arthralgia, cytopenia, hypocomplementemia, and lupus-specific antibodies supported active SLE (13), while antimitochondrial antibody (AMA) positivity and hypergammaglobulinemia prompted evaluation for concomitant autoimmune liver disease.

Prednisolone 50 mg/day was initiated on 14 June 2024 and maintained through 27 June. The dose was reduced to 40 mg/day on 28 June and subsequently tapered to 5 mg/day by 12 August. Intravenous belimumab was initiated on 24 June at 600 mg per infusion (10 mg/kg), every 2 weeks for the first three doses and every 4 weeks thereafter. Ursodeoxycholic acid (UDCA, 250 mg three times daily) was administered from 20 May 2024 for the cholestatic component. Supportive measures included albumin and fibrinogen supplementation. Aminotransferases had already declined before belimumab initiation, and subsequent treatment was accompanied by further biochemical improvement and resolution of anorexia and ascites (**Figure 1**; **Supplementary Tables S1, S2**). Coagulation indices normalized, enabling percutaneous liver biopsy.

**Figure 1:**
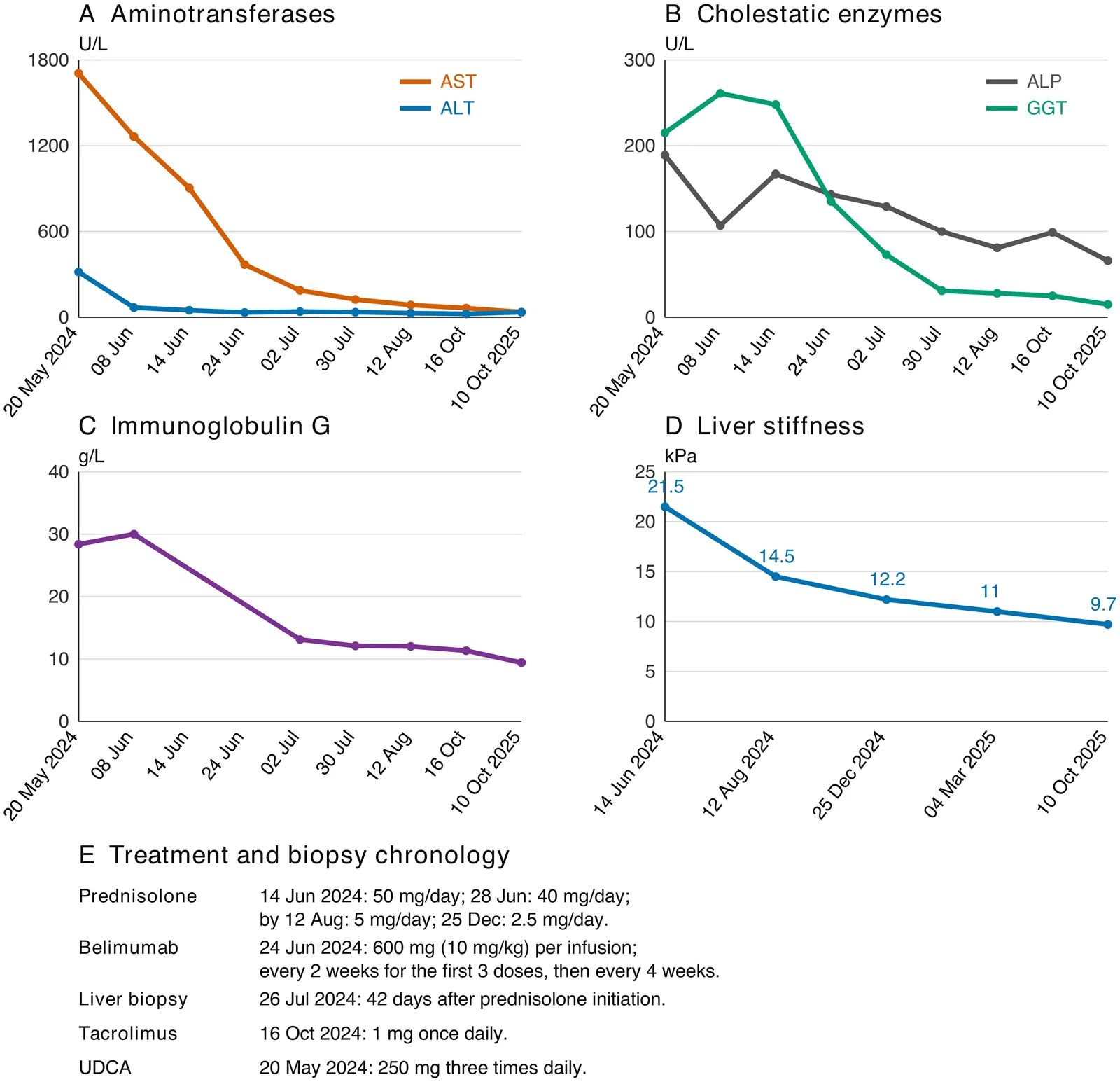
Longitudinal laboratory measurements, liver stiffness, and treatment chronology. **(A)** AST and ALT. **(B)** ALP and GGT. **(C)** IgG. **(D)** Liver stiffness measured by transient elastography. **(E)** Treatment initiation, corticosteroid dose reductions, and liver biopsy. Biopsy was performed on 26 July 2024, 42 days after prednisolone initiation. Sampling visits are displayed at equal spacing. Dated measurements and treatment details are provided in **Supplementary Tables S1, S2**. AST, aspartate aminotransferase; ALT, alanine aminotransferase; ALP, alkaline phosphatase; GGT, gamma-glutamyltransferase; IgG, immunoglobulin G; UDCA, ursodeoxycholic acid.

Liver biopsy was performed on 26 July 2024, 42 days after prednisolone initiation and 32 days after the first belimumab infusion. Histology demonstrated portal-based lymphoplasmacytic inflammation with moderate-to-marked interface hepatitis and destructive small-bile-duct injury (**Figures 2A, B**). Masson’s trichrome showed portal and periportal fibrosis (**Figure 2C**). Fibrosis was classified as Batts–Ludwig stage 2, without bridging fibrosis or cirrhosis (14). Cluster of differentiation 38 (CD38) and multiple myeloma oncogene 1 (MUM1) immunostaining highlighted increased plasma cells within portal tracts (**Figures 2D, E**), and reticulin staining demonstrated the portal architectural changes (**Figure 2F**). Together with AMA-M2 positivity, hypergammaglobulinemia, and mixed hepatitic and cholestatic biochemical abnormalities, these findings supported a clinicopathologic diagnosis of AIH–PBC overlap. Based on the local laboratory upper limits of normal (ULNs) of 40 U/L for ALT, 45 U/L for GGT, and 16 g/L for IgG, ALT was 7.93 × ULN, GGT 5.80 × ULN, and IgG 1.88 × ULN. The ALT and interface-hepatitis criteria supported the AIH component; the GGT and AMA criteria supported the PBC component (15, 16). The IgG/SMA criterion was not met. Thus, two criteria for each component were met (**Table 1**).

**Figure 2:**
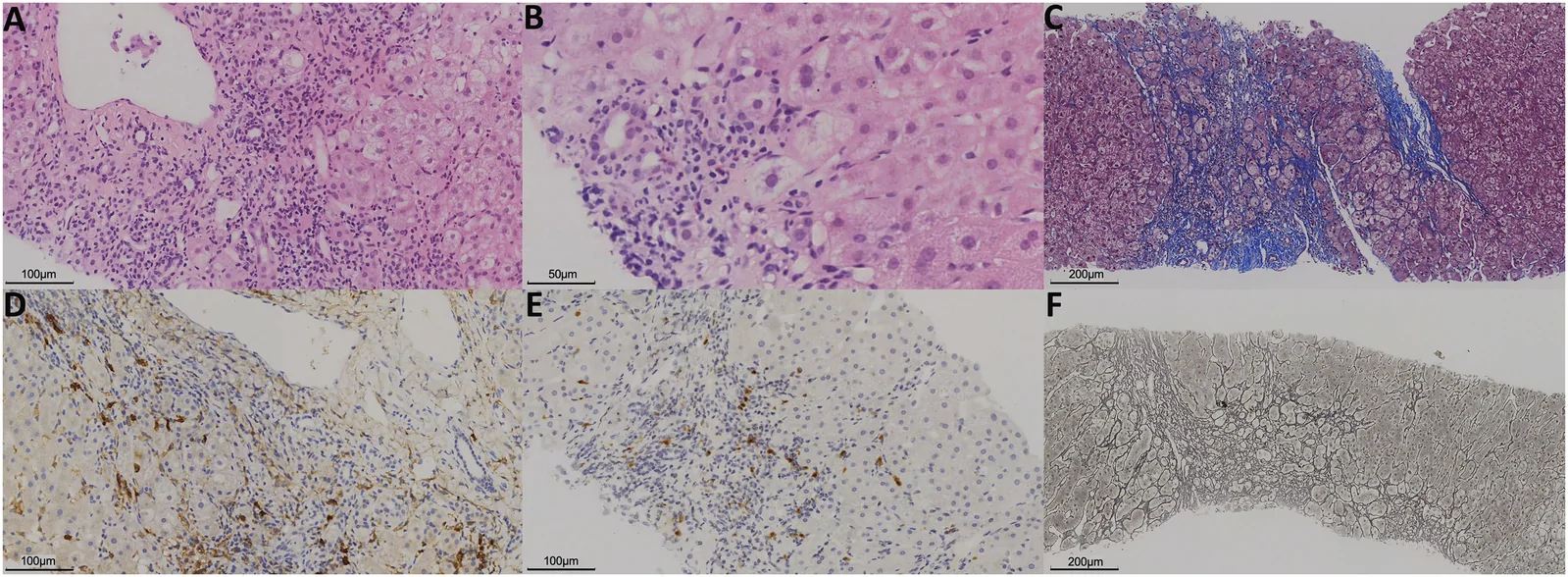
Liver biopsy obtained on 26 July 2024, 42 days after prednisolone initiation and 32 days after the first belimumab infusion. **(A, B)** Portal-based lymphoplasmacytic inflammation with interface hepatitis and destructive small-bile-duct injury, hematoxylin and eosin. **(C)** Portal and periportal fibrosis, Masson’s trichrome. **(D, E)** Plasma cells highlighted by CD38 and MUM1 immunostaining, respectively. **(F)** Portal architectural changes, reticulin stain. Fibrosis was classified as Batts–Ludwig stage 2 without bridging fibrosis or cirrhosis. Scale bars: A, 100 μm; B, 50 μm; C, 200 μm; D, 100 μm; E, 100 μm; F, 200 μm. CD38, cluster of differentiation 38; MUM1, multiple myeloma oncogene 1.

**Table 1:** Paris criteria for AIH–PBC overlap.

Domain	Criterion	Patient finding	Assessment
AIH	ALT ≥5 × ULN	ALT 317 U/L; local ULN 40 U/L; 7.93 × ULN	Met
AIH	IgG ≥2 × ULN or SMA positive	IgG 30.02 g/L; local ULN 16 g/L; 1.88 × ULN; SMA negative	Not met
AIH	Moderate or severe interface hepatitis	Moderate-to-marked interface hepatitis	Present on post-treatment biopsy
PBC	ALP ≥2 × ULN or GGT ≥5 × ULN	Peak GGT 261 U/L; local ULN 45 U/L; 5.80 × ULN	Met by GGT
PBC	AMA positive	AMA-M2 36.9 RU/mL, reported positive	Present
PBC	Florid duct lesion	Destructive small-bile-duct injury	Supportive; florid duct lesion not specifically established

Local laboratory ULNs were ALT 40 U/L, GGT 45 U/L, and IgG 16 g/L. Before immunosuppression, peaks were ALT 317 U/L (20 May 2024) and GGT 261 U/L (8 June 2024). AIH met two criteria and PBC met two criteria. The IgG/SMA criterion was not met. Destructive bile-duct injury was not counted as a confirmed florid duct lesion. AST is not part of the Paris criteria. AIH, autoimmune hepatitis; ALP, alkaline phosphatase; ALT, alanine aminotransferase; AMA-M2, antimitochondrial M2 antibody; AST, aspartate aminotransferase; GGT, gamma-glutamyltransferase; IgG, immunoglobulin G; PBC, primary biliary cholangitis; ULN, upper limit of normal; SMA, smooth muscle antibody; AMA, antimitochondrial antibody.

Tacrolimus 1 mg once daily was introduced on 16 October 2024 for maintenance immunosuppression. Prednisolone was reduced from 5 to 2.5 mg/day on 25 December 2024. Belimumab and UDCA were continued. At the last follow-up on 10 October 2025, the patient remained clinically well without reported relapse. Liver biochemistry was near-normal (ALT 36 U/L, AST 37 U/L, alkaline phosphatase [ALP] 66 U/L, GGT 15 U/L), with albumin 45 g/L, IgG 9.42 g/L, and IgG4 0.34 g/L. Antiphospholipid antibodies became negative. Liver stiffness measurement (LSM), assessed by transient elastography (FibroScan; Echosens SA, Paris, France), decreased from 21.5 kPa on 14 June 2024 to 9.7 kPa on 10 October 2025 (**Figure 1**).

## Discussion

This case illustrates the coexistence of systemic and organ-specific autoimmunity: new-onset SLE with severe acute hepatic dysfunction and biopsy findings supporting AIH–PBC overlap. Coagulopathy, ascites, hepatic encephalopathy, marked hypergammaglobulinemia, lupus-specific serologies, AMA positivity, and interface hepatitis with small-bile-duct injury supported an autoimmune liver disease phenotype. Sustained biochemical improvement occurred during corticosteroid induction with early adjunctive belimumab and UDCA, followed by tacrolimus maintenance.

Liver test abnormalities are relatively common in SLE, but severe hepatic dysfunction should not be automatically attributed to lupus hepatitis. Lupus hepatitis is usually mild; severe or progressive hepatitis warrants evaluation for coexisting autoimmune liver disease, viral infection, drug-induced injury, metabolic disease, and vascular injury (2–4). In this patient, interface hepatitis with plasma cell-rich inflammation and destructive small-bile-duct injury supported concomitant AIH–PBC overlap. An INR of 1.8 and hepatic encephalopathy supported an acute liver failure presentation, but Batts–Ludwig stage 2 fibrosis established pre-existing structural injury. There was no histological cirrhosis, although an acute presentation of previously unrecognized chronic autoimmune liver disease remained possible. Ascites and thrombocytopenia raised concern for underlying chronic liver disease but did not, in isolation, establish cirrhosis or chronic decompensation. Neither a previously normal liver panel nor the early LSM alone resolves this distinction.

AIH–PBC overlap remains diagnostically challenging. The Paris criteria require at least two of three features for each component, with moderate or severe interface hepatitis central to the AIH component (15, 16). AST is not a Paris criterion, and SMA negativity does not exclude AIH. In this case, ALT and GGT exceeded the required multiples using the local laboratory ULNs, whereas IgG did not reach 2 × ULN. Destructive small-bile-duct injury was supportive but was not counted as a confirmed florid duct lesion. A cohort of patients treated for AIH–PBC showed that many fell outside Paris criteria (17), supporting an integrated clinicopathologic assessment (18).

European Association for the Study of the Liver (EASL) guidance supports corticosteroid induction for active AIH and individualized maintenance immunosuppression, with calcineurin inhibitors among the options for selected patients (19). UDCA remains the basis of PBC treatment, with immunosuppression added when significant interface hepatitis is present (20). Belimumab was introduced 10 days after prednisolone in the setting of active SLE, and tacrolimus was added several months later. This sequence is important: the early biochemical improvement preceded tacrolimus and had begun before belimumab, so improvement cannot be attributed specifically to BAFF blockade.

Marked hypergammaglobulinemia, multiple autoantibodies, and plasma cell-rich hepatic inflammation provide a biological rationale for considering the B-cell pathway. BAFF promotes autoreactive B-cell survival, and serum BAFF and interleukin-21 (IL-21) have been associated with treatment response in AIH (11). However, BAFF concentrations and circulating or tissue B-cell subsets were not measured in this patient. These clinical and histological features therefore do not establish BAFF dependence, a validated B-cell-dominant subtype, or a biomarker for selecting belimumab.

Clinical evidence for BAFF blockade in autoimmune liver disease remains limited. Two refractory AIH patients achieved biochemical responses with add-on belimumab (21), and a subsequent small series reported improvement in selected difficult-to-treat AIH cases without a consistent biochemical effect in PBC (12). Reports in lupus hepatitis and AIH–SLE overlap provide additional observations (22, 23), while severe acute hepatic presentations in SLE have also been described with conventional treatment (24, 25). The present case adds a longitudinal observation in an AIH–PBC overlap phenotype, but it cannot establish efficacy, comparative safety, or treatment-selection criteria. Reports involving rituximab illustrate another B-cell-directed approach in overlap disease and difficult-to-treat AIH (6, 26); they do not establish an advantage of belimumab over B-cell depletion.

Although serum IgG4 was elevated, liver tissue showed fewer than 10 IgG4-positive plasma cells per high-power field and an IgG4+/IgG+ plasma cell ratio below 10%. These findings did not support IgG4-related hepatopathy (27, 28). The biopsy was obtained after 42 days of corticosteroid treatment and 32 days of belimumab exposure. Prior immunosuppression may have attenuated portal/interface inflammation and plasma-cell infiltration; the biopsy therefore does not represent untreated inflammatory activity (19). Treatment-related changes in inflammation cannot establish a change in fibrosis stage, and no repeat biopsy was obtained.

LSM decreased from 21.5 to 9.7 kPa over approximately 16 months. The initial measurement was obtained during active hepatitis. In AIH, early LSM is influenced by inflammation, whereas measurements after at least 6 months of treatment more reliably distinguish advanced fibrosis and cirrhosis (29). Follow-up LSM also has prognostic value in AIH (30), and elastography has been studied in AIH–PBC overlap (31). These findings support its use for longitudinal monitoring, but improved staging performance later in treatment does not prove that the fall from an inflamed baseline represents fibrosis regression. Without paired histology, the contributions of reduced inflammation, cholestatic injury, and any change in fibrosis cannot be separated.

Several limitations remain. The sequential and overlapping use of corticosteroids, UDCA, belimumab, and tacrolimus prevents attribution of benefit to any single drug. The post-treatment biopsy may underestimate initial inflammation. The full acute viral-hepatitis work-up and herbal/supplement exposure history could not be completely characterized. The marked AST elevation and antiphospholipid antibody positivity also warranted assessment for ischemic, muscle, and hepatic vascular causes; the available work-up did not permit their definitive exclusion. Tacrolimus trough concentrations and longitudinal renal, electrolyte, blood-pressure, and infection-monitoring results were unavailable, limiting assessment of treatment safety. Repeat histology was not performed.

In summary, severe hepatic dysfunction in SLE warrants assessment for concomitant autoimmune liver disease. Liver biopsy supported an AIH–PBC overlap phenotype while identifying pre-existing fibrosis and the limitations of post-treatment sampling. Sustained biochemical improvement during a belimumab-containing multidrug regimen is hypothesis-generating and does not establish the independent efficacy of BAFF blockade or histological fibrosis regression.

## Patient perspective

The patient reported that her symptoms gradually improved during treatment and that she was able to return to her usual daily life. She expressed gratitude for the care she had received.

## Data Availability

The original contributions presented in the study are included in the article/**Supplementary Material**, further inquiries can be directed to the corresponding author/s.

## References

[1] Sohal A Nikzad N Kowdley KV . Overlap syndromes in autoimmune liver disease: a review. Transl Gastroenterol Hepatol. (2025) 10:33. doi: 10.21037/tgh-24-140 40337770 PMC12056124

[2] Gonzalez-Regueiro JA Cruz-Contreras M Merayo-Chalico J Barrera-Vargas A Ruiz-Margain A Campos-Murguia A . Hepatic manifestations in systemic lupus erythematosus. Lupus. (2020) 29:813–24. doi: 10.1177/0961203320923398 32390496

[3] Chowdhary VR Crowson CS Poterucha JJ Moder KG . Liver involvement in systemic lupus erythematosus: case review of 40 patients. J Rheumatol. (2008) 35:2159–64. doi: 10.3899/jrheum.080336 18793002

[4] Zhang L Yin L Lv W Wang Y Liu Y Gou C . Clinical analysis of patients with systemic lupus erythematosus complicated with liver failure. Clin Rheumatol. (2023) 42:1545–53. doi: 10.1007/s10067-023-06524-9 36795333 PMC10202995

[5] González LA Orrego M Ramírez LA Vásquez G . Primary biliary cirrhosis/autoimmune hepatitis overlap syndrome developing in a patient with systemic lupus erythematosus: a case report and review of the literature. Lupus. (2011) 20:108–11. doi: 10.1177/0961203310378673 20724352

[6] Wang CR Tsai HW Wu IC . Autoimmune hepatitis and primary biliary cholangitis overlap disease in a systemic lupus erythematosus patient receiving long-term B-cell depletion therapy. J Formos Med Assoc. (2023) 122:1219–20. doi: 10.1016/j.jfma.2023.05.005 37202236

[7] Das D Dixit VK . Overlap syndrome of autoimmune hepatitis, primary biliary cholangitis leading to decompensated cirrhosis in a patient of systemic lupus erythematosus: a case report of complex concomitant autoimmune disease. J Clin Exp Hepatol. (2022) 12:S17. doi: 10.1016/j.jceh.2022.07.05942574925

[8] Ghanimeh MA Likhitsup A Bearden A Kaddourah O Tamimi T . Overlap syndrome of autoimmune hepatitis, primary biliary cholangitis, and primary sclerosing cholangitis in a patient with systemic lupus erythematosus: a case report of complex autoimmune liver disease. Am J Gastroenterol. (2016) 111:S902–3. doi: 10.14309/00000434-201610001-01886

[9] Efe C Purnak T Ozaslan E Ozbalkan Z Karaaslan Y Altiparmak E . Autoimmune liver disease in patients with systemic lupus erythematosus: a retrospective analysis of 147 cases. Scand J Gastroenterol. (2011) 46:732–7. doi: 10.3109/00365521.2011.558114 21348808

[10] Efe C Wahlin S Ozaslan E Berlot AH Purnak T Muratori L . Autoimmune hepatitis/primary biliary cirrhosis overlap syndrome and associated extrahepatic autoimmune diseases. Eur J Gastroenterol Hepatol. (2012) 24:531–4. doi: 10.1097/MEG.0b013e328350f95b 22465972

[11] Biewenga M Heidt S Vergunst M Marijnissen CMJ de Man RA van der Eijk AA . B-cell activating factor and IL-21 levels predict treatment response in autoimmune hepatitis. JHEP Rep. (2022) 4:100460. doi: 10.1016/j.jhepr.2022.100460 35368991 PMC8971938

[12] Kolev M Sarbu AC Moller B Maurer B Kollert F Semmo N . Belimumab treatment in autoimmune hepatitis and primary biliary cholangitis - a case series. J Transl Autoimmun. (2023) 6:100189. doi: 10.1016/j.jtauto.2023.100189 36718275 PMC9883290

[13] Aringer M Costenbader K Daikh D Brinks R Mosca M Ramsey-Goldman R . 2019 European League Against Rheumatism/American College of Rheumatology classification criteria for systemic lupus erythematosus. Arthritis Rheumatol. (2019) 71:1400–12. doi: 10.1002/art.40930 31385462 PMC6827566

[14] Batts KP Ludwig J . Chronic hepatitis. An update on terminology and reporting. Am J Surg Pathol. (1995) 19:1409–17. doi: 10.1097/00000478-199512000-00007 7503362

[15] Chazouillères O Wendum D Serfaty L Montembault S Rosmorduc O Poupon R . Primary biliary cirrhosis-autoimmune hepatitis overlap syndrome: clinical features and response to therapy. Hepatology. (1998) 28:296–301. doi: 10.1002/hep.510280203 9695990

[16] Kuiper EMM Zondervan PE van Buuren HR . Paris criteria are effective in diagnosis of primary biliary cirrhosis and autoimmune hepatitis overlap syndrome. Clin Gastroenterol Hepatol. (2010) 8:530–4. doi: 10.1016/j.cgh.2010.03.004 20304098

[17] Stoelinga AEC Biewenga M Drenth JPH Verhelst X van der Meer AJP de Boer YS . Diagnostic criteria and long-term outcomes in AIH-PBC variant syndrome under combination therapy. JHEP Rep. (2024) 6:101088. doi: 10.1016/j.jhepr.2024.101088 38974367 PMC11225825

[18] Qin X Jin Z . Progress and perspectives in overlapping primary biliary cholangitis and autoimmune hepatitis: a comprehensive review. J Gastrointestin Liver Dis. (2025) 34:241–9. doi: 10.15403/jgld-6138 40580520

[19] European Association for the Study of the Liver . EASL Clinical Practice Guidelines on the management of autoimmune hepatitis. J Hepatol. (2025) 83:453–501. doi: 10.1016/j.jhep.2025.03.017 40348684

[20] Lindor KD Bowlus CL Boyer J Levy C Mayo M . Primary biliary cholangitis: 2021 practice guidance update from the American Association for the Study of Liver Diseases. Hepatology. (2022) 75:1012–3. doi: 10.1002/hep.32117 34431119

[21] Arvaniti P Giannoulis G Gabeta S Zachou K Koukoulis GK Dalekos GN . Belimumab is a promising third-line treatment option for refractory autoimmune hepatitis. JHEP Rep. (2020) 2:100123. doi: 10.1016/j.jhepr.2020.100123 32671332 PMC7340979

[22] Psenak O Studnicka-Benke A Haufe H Greil R . Good response of lupus hepatitis to mycophenolate mofetil and belimumab as of lupus exanthema to rituximab: A case report. Rheumatol: Curr Res. (2019) 9:247. doi: 10.35248/2161-1149.19.9.247

[23] Duggal S Sairam S . Role of B-cell inhibition in autoimmune hepatitis: lessons learnt from systemic lupus erythematosus. Cureus. (2025) 17:e81108. doi: 10.7759/cureus.81108 40276396 PMC12018065

[24] Mustafa M Bawazir YM . Acute liver failure as the first feature of systemic lupus erythematosus. Rheumatol Int. (2021) 41:469–74. doi: 10.1007/s00296-020-04717-y 33034712

[25] Singh AK Vaithiyam V Sonika U Goyal S . Severe acute liver injury: first manifestation of systemic lupus erythematosus. J Clin Exp Hepatol. (2024) 14:101339. doi: 10.1016/j.jceh.2023.101339 38264573 PMC10801300

[26] Riveiro-Barciela M Barreira-Diaz A Esteban P Rota R Alvarez-Navascues C Perez-Medrano I . Rituximab is a safe and effective alternative treatment for patients with autoimmune hepatitis: Results from the ColHai registry. Liver Int. (2024) 44:2303–14. doi: 10.1111/liv.15970 38809086

[27] Arase Y Matsumoto K Anzai K Tsuruya K Sugiyama S Yoshihara S . Clinicopathological features of autoimmune hepatitis with IgG4-positive plasma cell infiltration. Dig Dis. (2021) 39:225–33. doi: 10.1159/000510562 32731217 PMC8117379

[28] Tanaka A Notohara K Tobari M Abe M Umemura T Takahashi A . A clinicopathological study of IgG4-related autoimmune hepatitis and IgG4-hepatopathy. J Gastroenterol. (2025) 60:632–40. doi: 10.1007/s00535-025-02221-3 39921744 PMC12014833

[29] Hartl J Denzer U Ehlken H Zenouzi R Peiseler M Sebode M . Transient elastography in autoimmune hepatitis: Timing determines the impact of inflammation and fibrosis. J Hepatol. (2016) 65:769–75. doi: 10.1016/j.jhep.2016.05.023 27238753

[30] Olivas I Arvaniti P Gabeta S Torres S Del Barrio M Diaz-Gonzalez A . Liver stiffness measurement predicts clinical outcomes in autoimmune hepatitis. JHEP Rep. (2024) 6:101213. doi: 10.1016/j.jhepr.2024.101213 39524208 PMC11550196

[31] Wang X Li Y Jiang L Zhou M Zhang X Wen H . Performance of 2D-shear wave elastography in autoimmune hepatitis-primary biliary cholangitis overlap syndrome. Abdom Radiol (NY). (2023) 48:1290–7. doi: 10.1007/s00261-023-03840-1 36792725

