# Supplementary material for: Case Report: Acute liver failure in systemic lupus erythematosus with autoimmune hepatitis–primary biliary cholangitis overlap: a belimumab-containing regimen and longitudinal follow-up
Source: Front Immunol. 2026 Oct 6;17:1915385. doi: 10.3389/fimmu.2026.1915385 (PMC13680148; doi:10.3389/fimmu.2026.1915385)
Supplement: Supplementary file 1 [file Table1.docx]

Supplementary material

Case Report: Acute liver failure in systemic lupus erythematosus with autoimmune hepatitis–primary biliary cholangitis overlap: a belimumab-containing regimen and longitudinal follow-up

# Supplementary Table S1 Longitudinal clinical measurements

Values are reported at the available sampling dates. A dash denotes a measurement that is not available, not a normal result.

| **Date** | **AST U/L** | **ALT U/L** | **ALP U/L** | **GGT U/L** | **Bilirubin μmol/L** | **Albumin g/L** |
| --- | --- | --- | --- | --- | --- | --- |
| 20 May 2024 | 1706 | 317 | 189 | 215 | 30.9 | 32.5 |
| 08 Jun 2024 | 1263 | 68 | 107 | 261 | 36.4 | 27.4 |
| 14 Jun 2024 | 904 | 49 | 167 | 248 | 45.1 | 31.5 |
| 24 Jun 2024 | 369 | 34 | 143 | 135 | 31.7 | 26.4 |
| 02 Jul 2024 | 188 | 40 | 129 | 73 | 27.2 | 34.9 |
| 30 Jul 2024 | 125 | 36 | 100 | 31 | 13.9 | 32.5 |
| 12 Aug 2024 | 86 | 29 | 81 | 28 | 12.4 | 34.7 |
| 16 Oct 2024 | 64 | 24 | 99 | 25 | 11 | 41.4 |
| 10 Oct 2025 | 37 | 36 | 66 | 15 | — | 45 |

AST, aspartate aminotransferase; ALT, alanine aminotransferase; ALP, alkaline phosphatase; GGT, gamma-glutamyltransferase.

## Immunoglobulins and liver stiffness

| **Date** | **IgG g/L** | **IgG4 g/L** |
| --- | --- | --- |
| 20 May 2024 | 28.39 | 4.21 |
| 08 Jun 2024 | 30 | — |
| 24 Jun 2024 | — | 7.21 |
| 02 Jul 2024 | 13.11 | — |
| 30 Jul 2024 | 12.09 | 1.61 |
| 12 Aug 2024 | 12.01 | — |
| 16 Oct 2024 | 11.33 | 0.5 |
| 10 Oct 2025 | 9.42 | 0.34 |

Peak IgG was 30.02 g/L, rounded to 30 g/L in the longitudinal plot.

| **Date** | **Liver stiffness kPa** |
| --- | --- |
| 14 Jun 2024 | 21.5 |
| 12 Aug 2024 | 14.5 |
| 25 Dec 2024 | 12.2 |
| 04 Mar 2025 | 11 |
| 10 Oct 2025 | 9.7 |

# Supplementary Table S2 Treatment milestones

| **Date** | **Intervention or assessment** |
| --- | --- |
| 20 May 2024 | UDCA 250 mg three times daily initiated |
| 14 Jun 2024 | Prednisolone 50 mg/day initiated; maintained through 27 June |
| 24 Jun 2024 | Belimumab 600 mg (10 mg/kg) per infusion initiated; every 2 weeks for the first three doses, then every 4 weeks |
| 28 Jun 2024 | Prednisolone reduced to 40 mg/day |
| 26 Jul 2024 | Liver biopsy, 42 days after corticosteroid initiation |
| 12 Aug 2024 | Prednisolone had been reduced to 5 mg/day |
| 16 Oct 2024 | Tacrolimus 1 mg once daily initiated |
| 25 Dec 2024 | Prednisolone reduced to 2.5 mg/day |
| 10 Oct 2025 | Latest clinical follow-up; prednisolone 2.5 mg/day, continued belimumab, tacrolimus, and UDCA |
